# Supplementary material for: Upstream open reading frame inactivation augments GATA4 translation and cardiomyocyte hypertrophy in mice
Source: bioRxiv. 2025 May 18:2025.05.18.654700. Preprint. [Version 1] doi: 10.1101/2025.05.18.654700 (PMC12132206; doi:10.1101/2025.05.18.654700)
Supplement: 1 [file NIHPP2025.05.18.654700V1-supplement-1.pdf]

bioRxiv preprint doi: https://doi.org/10.1101/2025.05.18.654700; this version posted May 18, 2025. The copyright holder for this preprint (which was not certified by peer review) is the author/funder, who has granted bioRxiv a license to display the preprint in perpetuity. It is made available under aCC-BY 4.0 International license.

Supplementary information

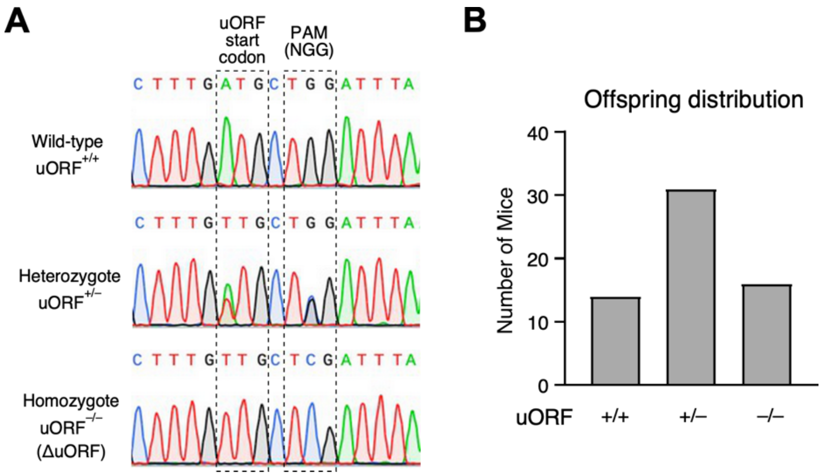

Supplementary Figure 1. Generation and characterization of *Gata4* ΔuORF mice

A. Electropherogram displaying DNA sequences obtained from toe clippings of mice with wild-type, heterozygous, and homozygous genotypes, visualized in SnapGene software. **B**. Mendelian distribution of offspring (N = 61; 14 +/+, 31 +/–, 16 –/–) resulting from the breeding of heterozygous mice adheres to the expected 1: 2: 1 ratio of wild-type, heterozygous, and homozygous mice.

bioRxiv preprint doi: https://doi.org/10.1101/2025.05.18.654700; this version posted May 18, 2025. The copyright holder for this preprint (which was not certified by peer review) is the author/funder, who has granted bioRxiv a license to display the preprint in perpetuity. It is made available under aCC-BY 4.0 International license.

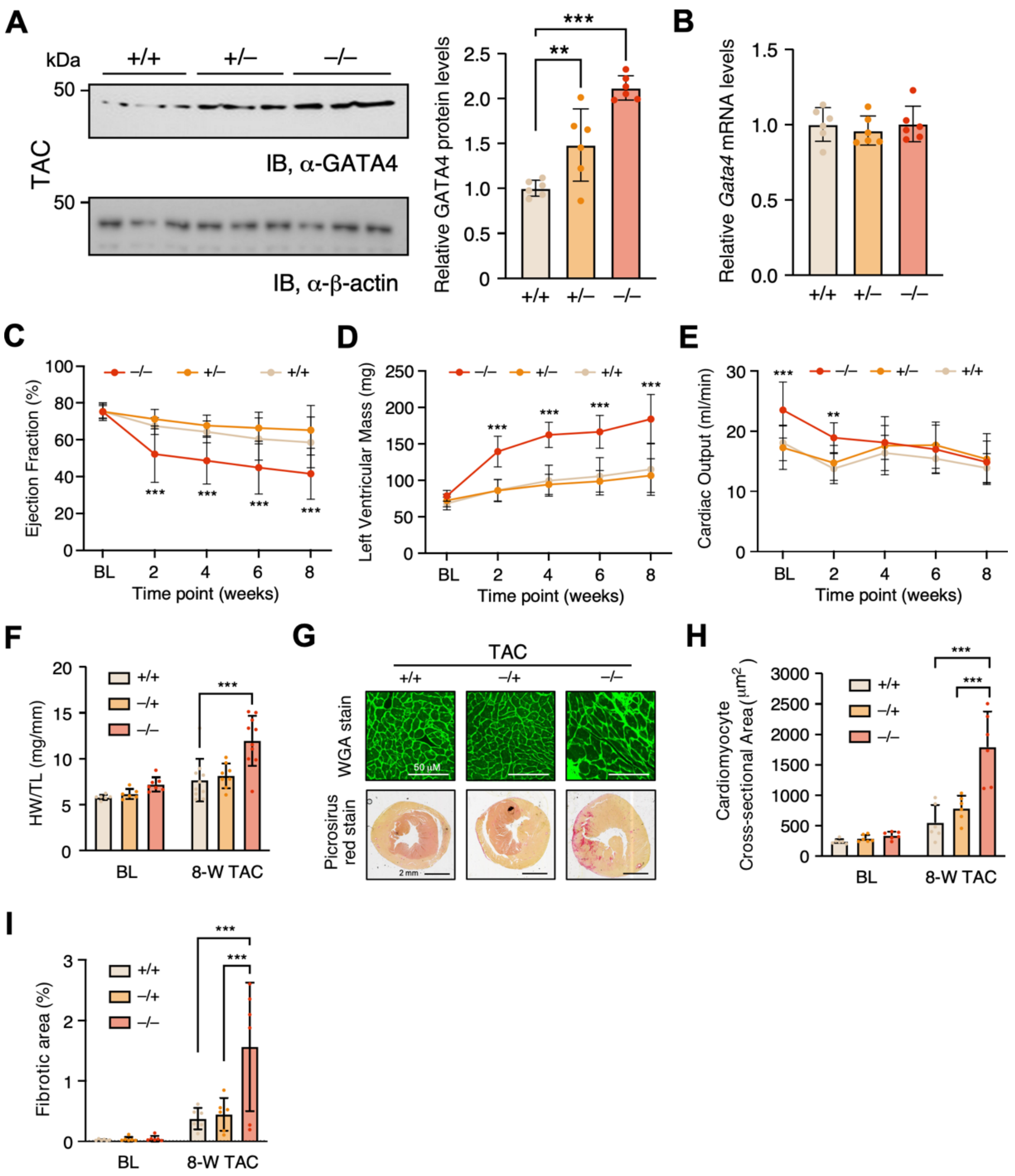

Supplementary Figure 2. Gata4 ΔuORF mice show increased protein levels and lead to cardiac decompensation in response to transverse aortic constriction surgery.

A. Western blotting showing the protein expression of GATA4 in wild-type (WT), heterozygous, and homozygous heart lysates normalized to β-actin followed by quantification. **B**. RT-qPCR of *Gata4* mRNA normalized by *Actb* for samples in **A**. **C-E**. Echocardiography parameters over 8 weeks for the mice subjected to TAC surgery. **F-I**.

bioRxiv preprint doi: https://doi.org/10.1101/2025.05.18.654700; this version posted May 18, 2025. The copyright holder for this preprint (which was not certified by peer review) is the author/funder, who has granted bioRxiv a license to display the preprint in perpetuity. It is made available under aCC-BY 4.0 International license.

characterizing hypertrophy and fibrosis of the isolated hearts at the 8-week endpoint. **F**. Heart weight normalized to the tibia length as an indicator of hypertrophy. **G**. Upper: Immunomicrograph of mouse cross-sections stained with wheat germ agglutinin (WGA)-Alexa fluor 480 to highlight cellular cross-sectional area, quantified in **H**. Lower: Scan of heart tissue slides stained with picrosirius red, which highlights collagen deposition, quantified in **I**. Data are represented as mean ± SD. ** $P < 0.05$, ** $P < 0.01$, *** $P < 0.001$; Statistical significance was confirmed by 2-way ANOVA followed by Holm-Sidak post hoc test.

bioRxiv preprint doi: https://doi.org/10.1101/2025.05.18.654700; this version posted May 18, 2025. The copyright holder for this preprint (which was not certified by peer review) is the author/funder, who has granted bioRxiv a license to display the preprint in perpetuity. It is made available under aCC-BY 4.0 International license.

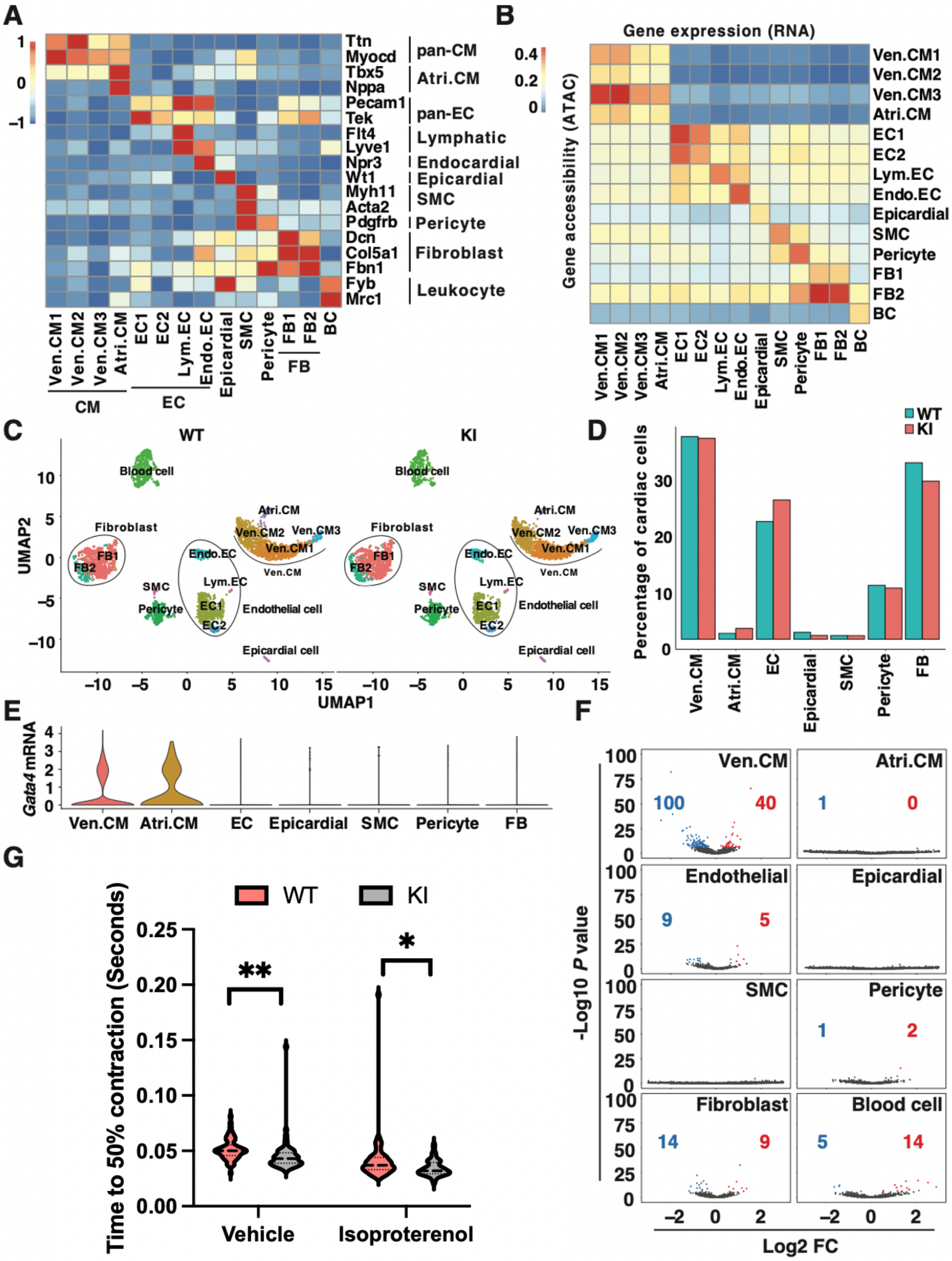

Supplementary Figure 3. snRNA/ATAC-seq analysis of wildtype and ΔuORF KI mouse hearts.

A. A heatmap showing the gene accessibilities (summarized chromatin accessibility of promoter and gene body) of marker genes as shown in the Violin Plots in Figure 4C.

bioRxiv preprint doi: https://doi.org/10.1101/2025.05.18.654700; this version posted May 18, 2025. The copyright holder for this preprint (which was not certified by peer review) is the author/funder, who has granted bioRxiv a license to display the preprint in perpetuity. It is made available under aCC-BY 4.0 International license.

Scaled and normalized accessibility were plotted. **B**. A heatmap showing the correlation between gene expressions and gene accessibilities within and among clusters. Only the top 3000 variant genes were used to calculate the correlation. **C**. A UMAP presentation of the clustering of wildtype and ΔuORF KI nucleus. **D**. A bar plot showing the percentage of each cardiac cell type in total cardiac cells in wildtype and ΔuORF KI hearts. Blood cells were excluded from the calculation. **E**. Violin Plots showing the *GATA4* mRNA expression in cardiac cells. Scaled and normalized expressions were plotted as the y-axis. **F**. Volcano plots showing the gene expression changes in ΔuORF hearts in each cell type. Genes with significantly increased expression in ΔuORF hearts (Log_2 fold change > 0.2 and Bonferroni correction adjusted *P*-value < 0.05) were red-colored. Genes with significantly decreased expression (Log_2 fold change < -0.2 and Bonferroni correction adjusted *P*-value < 0.05) in ΔuORF KI hearts were blue-colored. **G**. A bar graph represents the time cardiomyocytes need to reach 50% of their maximum contraction at baseline (without ISO treatment) and upon isoproterenol (ISO; 10 μM) stimulation.. Ven.CM, ventricular cardiomyocytes; Atri.CM, atrial cardiomyocytes; FB, fibroblasts; EC, endothelial cells; SMC, smooth muscle cells; BC, blood cells.

bioRxiv preprint doi: https://doi.org/10.1101/2025.05.18.654700; this version posted May 18, 2025. The copyright holder for this preprint (which was not certified by peer review) is the author/funder, who has granted bioRxiv a license to display the preprint in perpetuity. It is made available under aCC-BY 4.0 International license.

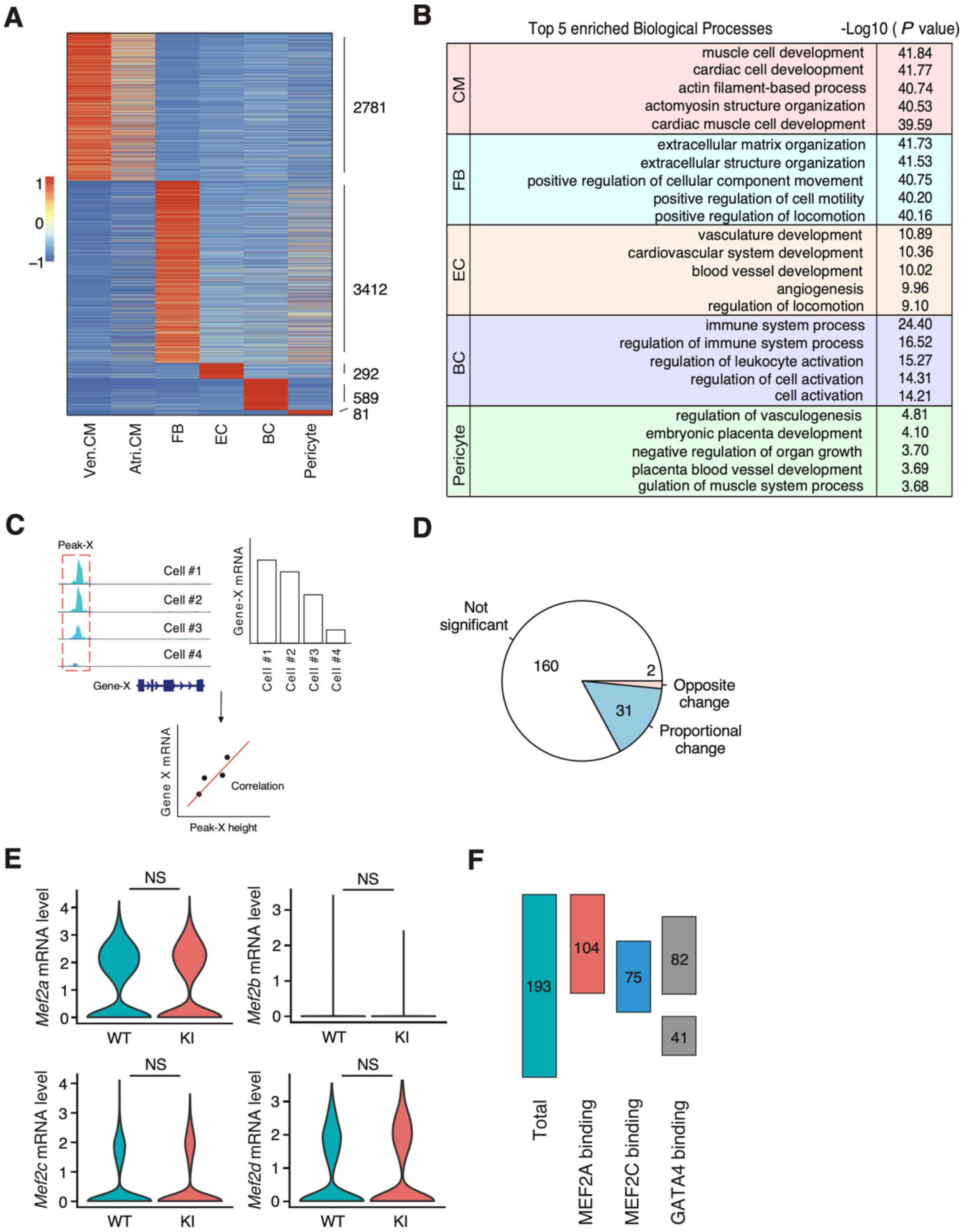

Supplementary Figure 4. Identification of cis-regulatory DNA elements with altered accessibility in ΔuORF ventricular cardiomyocytes.

A. A heatmap showing the accessible chromatin regions specific to CM, FB, EC, pericytes, and BC. Scaled and normalized accessibility were plotted. **B.** Genomic Regions

bioRxiv preprint doi: https://doi.org/10.1101/2025.05.18.654700; this version posted May 18, 2025. The copyright holder for this preprint (which was not certified by peer review) is the author/funder, who has granted bioRxiv a license to display the preprint in perpetuity. It is made available under aCC-BY 4.0 International license.

Enrichment of Annotations Tool (GREAT) analysis results showing the top 5 enriched biological processes of the cell type-specific regions in Figure S4A. **C**. A sketch showing the workflow of identifying region-gene correlated pairs. The expected coefficient values were calculated for each pair to compute a z-score and *P*-value. Pairs with $P < 0.05$ were considered as region-gene correlated pairs. The Chromatin region in those pairs is treated as pCREs of the correlated genes. **D**. A pie chart shows that gene changes correlated with significantly changed pCREs. 160 of the pCREs with significantly changed accessibility in ΔuORF Ven.CM correlated with genes that have not changed significantly (not significant). 31 of the pCREs change in the same direction as their correlated genes (both increase or decrease in ΔuORF Ven.CM) (coordinate change). 2 of the pCREs change in the opposite direction as their correlated genes (opposite change). **E**. Violin Plots showing the *Mef2a, Mef2b, Mef2c, Mef2d* mRNA expression in Ven.CM. Scaled and normalized expressions were plotted on the y-axis. **F**. A sketch showing the overlap of the significantly changed pCREs and published ChIP-seq data. 104 of the 193 pCREs overlap with MEF2A binding sites, 75 of the 193 pCREs overlap with MEF2C binding sites, and 123 of the 193 pCREs overlap with MEF2A binding sites. CM, cardiomyocytes; FB, fibroblasts; EC, endothelial cells; BC, blood cells.
